# A Case Study of Refined Building Climate Zoning under Complicated Terrain Conditions in China

**DOI:** 10.3390/ijerph19148530

**Published:** 2022-07-12

**Authors:** Tianyu Zhang, Xianyan Chen, Fen Zhang, Zhi Yang, Yong Wang, Yonghua Li, Linxiao Wei

**Affiliations:** 1 Chongqing Climate Center, Chongqing Meteorological Administration, Chongqing 401147, China; zhangtianyu821227@hotmail.com (T.Z.); zhangfen@mail.bnu.edu.cn (F.Z.); wngyng@hotmail.com (Y.W.); lyhcq@163.com (Y.L.); quiet7@126.com (L.W.); 2 National Climate Center, China Meteorological Administration, Beijing 100081, China; 3 Chongqing Meteorological Administration, Chongqing 401147, China; cqycyz@126.com

**Keywords:** ASHRAE Standard 169-2021, GB50176-2016, heating degree-days, cooling degree-days

## Abstract

In this study, we first found that the few and sparse meteorological stations used in earlier comprehensive studies of building climate zoning in a complicated terrain area like Chongqing, China, may lead to the inapplicability of building energy efficiency standards in some areas. To address this issue, the study used daily data from 1908 extremely dense surface meteorological stations from 2011 to 2020 in Chongqing, China. In order to conduct fine zoning of building climate in Chongqing, China, GB50176-2016 and ASHRAE standard 169-2021 were employed, respectively. The findings indicated that by using the ASHRAE standard, the entire Chongqing region was classified into five climate zones. The Chongqing region was categorized into three different climate zones using China GB50176-2016: cold zone (CZ), hot summer and cold winter zone (HSCWZ), and mild zone (MZ). Not to be overlooked is the MZ (China’s GB50176-2016)/mixed-humid zone (ASHRAE standard), which is primarily situated at higher elevations in the southeast and northeast of Chongqing. In comparison to the HSCWZ/warm-humid zone, these zones have drastically different building energy efficiency regulations and approaches. According to preliminary projections, improved building climate zoning will to some extent increase building energy efficiency and reduce emissions in Chongqing. Finally, this study case can be replicated in different regions with complicated terrain.

## 1. Introduction

China’s aims for carbon peaking and carbon neutrality led to new specifications for the low-carbon transformation of building and other industries. Scientific building climate zoning is a necessary prerequisite for the development of green buildings and the realization of reasonable architectural design. Building climate zoning, which reflects the fundamental relationship between buildings and climate, entails energy-saving climate-based design that provides macro-control and guidance for building planning, design, and construction in various areas [1,2]. Scientific building climate zoning can offer substantial technical assistance for the design of useful building energy conservation policies and standards in order to ascertain regional climatic characteristics, judiciously employ regional climate resources, and prevent undesirable climate consequences.

According to Walsh’s review of climate zoning methods for building energy efficiency projects in 54 countries, the temperature, degree-day, altitude, and relative humidity were found to be the most frequently used variables, techniques, and parameters for climate zoning, with up to three variables, techniques, or parameters being used in about 80% of countries [3]. Walsh verified performance-based climate zoning for applications related to building energy conservation [4], and Mazzaferro L investigated whether building performance data was required to construct climate zones for requirements related to building energy conservation [5]. The selection of zoning indicators varied significantly between different regions and nations, according to an analysis of 27 climate zoning systems for building energy efficiency conducted by Bai et al. in 2020 over 20 countries and regions [6]. There were observable disparities in the adopted zoning indicators, even if the aim of zoning was the same. In order to promote climate zoning for building energy policies, there is not enough research to demonstrate which type of zoning is the most correct [7].

Building climate zoning requirements vary between China and other nations. To identify building climate zones, Spain and Chile have both used a combination of winter and summer climatic severities (WCS and SCS, respectively) obtained from building technical codes [8,9,10]. In the 21st century, Europe has performed research on climate zoning schemes to guide the design of near-zero energy buildings [11]. Both the IECC [12] and ASHRAE in the U.S. have adopted the wet and dry secondary climate zoning scheme originally developed by Robert et al. [13,14].

The ASHRAE climate zoning criteria are regularly revised [15,16,17,18], and the zoning is changing, and the revisions reflect the influence of global warming on climate classification [19]. For design, planning, and the selection of building energy systems and equipment, ASHRAE climate zoning guidelines can offer the necessary climate information. In various nations and areas throughout the world, including Iran [20] and Ethiopia [21], ASHRAE building climate zoning regulations have been extensively examined and used in recent years. Using the cluster analysis method, Bai L et al. updated the findings of China’s building climate zoning based on ASHRAE standard 169-2013 [22]. The main distinction between the Chinese climate zoning method and the ASHRAE climate zoning standard is that the Chinese approach does not take administrative zones into account, whereas the ASHRAE standard simultaneously takes climatic elements into account [16,22,23].

In China, studies on climate zoning and architecture date all the way back to 1955 [24]. Numerous academics have recently carried out pertinent studies on building climate zoning in China. In China, climate zoning based on passive climate design, such as solar energy, was researched by Lam et al. and Lau et al. [25,26]. In order to study climate zoning for building energy efficiency in China, Fu X Z et al. employed HDD18, CDD26, and other indices [27]. Five bioclimatic areas were divided using cluster analysis by Wan K et al. using the summer and winter comfort index CI [28]. Based on main component cluster analysis, Zhang H.L. et al. carried out climate zoning for building energy efficiency in 270 Chinese cities [29].

The viability of several strategies in climate zoning for national building energy efficiency design was compared and examined by Bai et al. [30]. The effect of climate change on China’s thermal temperature zone and building energy regulations was examined by Bai et al. in 2019 [31]. In 2020, Liu et al. studied the shifts in China’s building climate zones between 1951 to 1980 and 1981 to 2020 [32]. Building climate zoning was investigated in China by Yang et al. using a machine learning-based method including supervised categorization [33]. The findings and methodology could help other nations and regions with building climate zoning. Additionally, other researchers have studied building climate zoning, taking into account a certain type of building climate zone and a specific area, such as the HSCWZ [34] and major ocean islands of China [35].

Chinese building climate zoning studies have generally been expanded in recent years based on the *Code for Thermal Design of Civil Buildings* (GB50176-2016) [36]. Based on *the Standard of Climatic Regionalization for Architecture* (GB50178-93), individual investigations were expanded [1]. It shows that the key zoning indicators of GB50176-2016 are broadly acknowledged by a large number of researchers. In order to direct building energy efficiency and green building initiatives, China’s major cities have adopted GB50176-2016, which primarily deals with winter insulation and summer thermal insulation. However, this standard code only gives climatic characteristics for 354 Chinese cities and towns, significantly fewer than the actual number of cities and towns, not to mention that it does not cover all of China’s administrative districts. Furthermore, the meteorological station data included in the GB50176-2016 standard dates from 1995 to 2004, and given the effects of climate change, it is urgently necessary to update this data. Therefore, it becomes vital to refine thermal zoning because it is manifestly unsuitable to use the same thermal design standards for buildings in locations with complicated and variable topography and climate.

In China’s southeast Sichuan-Chongqing Basin and upper sections of the Yangtze River, Chongqing is situated in a hilly and mountainous region. The topography of Chongqing is highly undulating and has complicated terrain characteristics. The three-dimensional climate characteristics are quite important, and the elevation of the entire city runs from 73.1 to 2797.0 m. Building meteorological factors clearly vary between several places in Chongqing, China [37]. Climate change in Chongqing has been shown to have a considerable impact on building energy consumption and architectural factors [37,38]. The objective of this study is to use more recent and dense observed meteorological data to analyze and determine the refined building climate zoning in Chongqing region with complicated terrain conditions. The refined zoning results from this study may indicate areas where the conclusions differ from those of earlier research and/or have been improved. The purpose of this article also includes a brief discussion of whether sophisticated building climate zoning aids in energy savings. This study will improve design indicators for regional building energy efficiency requirements and provide significant technical support for the growth of green buildings in Chongqing’s diverse regions against a backdrop of carbon neutrality and carbon peaking.

## 2. Data and Methods

### 2.1. Data

**Figure 1:**
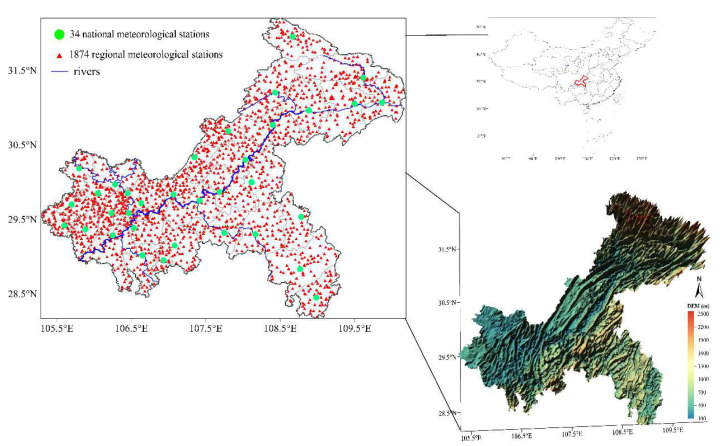
Geographical location of Chongqing in China, distribution of high-density meteorological stations in Chongqing, and digital elevation model (DEM) map of Chongqing (The green dots indicate 34 national meteorological stations, and the small red triangles indicate 1874 regional meteorological stations).

Chongqing is located in the southwestern part of China and the upper reaches of the Yangtze River. The Chongqing area spans the transition zone between the Qinghai–Tibet Plateau and the middle and lower reaches of the Yangtze River between 105°11′~110°11′ E and 28°10′~32°13′ N. With an area of 82,400 square kilometers, Chongqing belongs to the East Asian inland monsoon region [39]. The terrain in Chongqing is complex, with notable undulations, and the terrain gradually decreases from north to south towards the Yangtze River Valley. The southeastern portion is close to two significant mountain ranges, namely Daba Mountain and Wuling Mountain, while the northwestern and central portions are characterized by hills and low mountains. Platform and flat dam areas make up 3.6 percent and 2.4 percent of the total area, respectively, whereas mountainous areas make up 75.8 percent of the whole area and hilly areas make up 18.2 percent [40]. (Figure 1). 

By the end of 2021, there are 34 national meteorological stations and more than 2000 regional meteorological stations in Chongqing. The 34 national meteorological stations have complete observations of meteorological elements, and the data have been strictly quality-controlled [41], with a very high degree of completeness of daily data for temperature and precipitation. The missing rate of individual national meteorological stations is less than 1% every year for the 10 years between 2011 and 2020, which indicates that each station has at least 362 days of valid recorded data each year. Each regional meteorological station has temperature and precipitation observations, which are sufficient for this investigation, despite the regional meteorological stations having fewer observation elements than the national meteorological stations. We take into account both the utilization of as many local weather stations as possible and the accuracy of the data. The data from the local weather stations that passed our screening and met the criteria were used in this investigation. The requirement: Individual weather stations must have at least 347 days of valid recorded data every year with an annual temperature daily data deficiency rate of less than 5%. Finally, 1874 local weather stations were chosen. Finally, for the 10 years of 2011–2020, this study employed the daily temperature and precipitation data from 1874 regional meteorological stations and 34 national meteorological stations. The Chongqing Meteorological Bureau provided these statistics. The placement of 1908 national and local meteorological stations is depicted in Figure 1. There is a significant elevation difference in Chongqing. The regional meteorological stations (represented by the red triangles in Figure 1) are densely and fairly evenly distributed in various areas of Chongqing at various altitudes, in contrast to the 34 national meteorological stations (represented by the green dots in Figure 1), which are located at an altitude ranging from 166 to 800 m above sea level. There are 165 regional stations above 1000 m and 326 regional stations above 800 m, respectively, in high-altitude regions.

### 2.2. Method

First, a thorough explanation of the ASHREA requirements for building climate zoning was given. ASHRAE continued to revise and publish building climate zoning standards, from ASHRAE Standard 169-2006, Standard 169-2013, and Standard 169-2020 to the present Standard 169-2021 [15,16,17,18], using the climate zoning scheme created by Robert et al. [13,14]. The ASHRAE standard’s building climate zoning scheme divides the climate into three sorts, namely dry, wet, and oceanic climates, after first defining the climate type. Table 1 provides the mechanism for dividing climate types.

The HDD18 base temperature is 18.3 °C in the US according to the ASHRAE building climate zoning standard. This paper refers to the ASHRAE HDD18 standard as HDD18.3 to differentiate it from the Chinese standard. Following the determination of the climatic type, the aforementioned standard separates 9 regions based on the HDD18.3 and CDD10 values (Table 2) as well as the combination of the three climate types, producing a total of 19 climate regions (0A, 0B, 1A, 1B, 2A, 2B, 3A, 3B, 3C, 4A, 4B, 4C, 5A, 5B, 5C, 6A, 6B, 7, and 8). Each climate zone’s name conveys the matching dry and wet qualities as well as the corresponding cold and hot conditions. Walsh chose three states in the United States for his research areas and used building energy simulation and GIS-based performance assessment tools to verify the climate zones established by ASHRAE Standard 169-2013. The study found that approximately 10% of the data was incorrect, indicating a greater need for performance-based evaluation across all global climate subregions [7]. 

Second, the primary standards for China’s building climate zoning are introduced. The main primary zoning indicators in *the Code for thermal design of civil buildings* (GB50176-2016) were used for zoning in the study of building climate zoning in China conducted by Bai et al. [31], Cheng et al. [42], and Liu et al. (2020) [32]. Primary zoning indicators and design principles of China’s *Code for thermal design of civil buildings* (GB50176-2016) can be found in reference [36].

Finally, the following subsections provide a detailed explanation of the specific steps and methods used in this study:

(1) HDD18.3 and HDD18 indicate the variation in building heating demand as a result of the climate environment in different regions, and the indicators CDD10 and CDD26 reflect the variation in building cooling demand as a result of the climate environment in different regions [7,31]. In the literature, the precise calculation techniques of CDD26 and HDD18 (unit: °C·d) are presented [27,43]. Please consult the literature for the precise CDD10 and HDD18.3 calculation techniques (unit: °C·d) [18]. Prior to examining the refined building climate zoning in Chongqing, the revised spatial distribution features of HDD18, CDD26, and CDD10 utilizing the daily data of 1908 meteorological stations in Chongqing were calculated and compared. In the Chongqing area, the spatial variations in building cooling and heating demand were largely understood. Step 1 is the same as what is written in Section 3.1 of this essay. Step 1 corresponds to this paper’s Section 3.1.

(2) To conduct building climate zoning in Chongqing in accordance with ASHRAE Standard 169-2021, intensive meteorological station data (i.e., daily observation data of 1908 meteorological stations in Chongqing from 2011 to 2020) was used in this study instead of performance-based validation (ASHREA Standard 169-2021, ASHREA Standard 169-2021, ASHREA Standard 169-2020, and ASHREA Standard 169-2013 are the same in terms of climate zoning methods). This study’s zoning results and those provided by ASHRAE Standards are compared and assessed for similarities and discrepancies. Step 2 corresponds to this paper’s Section 3.2.

(3) Using data from dense meteorological stations, the main indicators of the primary zoning of the Chinese Thermal Design Standard for Civil Buildings (GB50176-2016) were used for building thermal design climate zoning in Chongqing. This study’s Chongqing zoning results were compared and evaluated, and the causes for the variations between them and those from previous research [30,31,32,33,36,42] were examined. The potential energy saving percentage of new public building construction was then originally evaluated based on China’s national standard *“General code for energy efficiency and renewable energy application in buildings”* (GB 55015-2021) [44] utilizing the modified zoning results of our study. Step 3 corresponds to Section 3.3 of this paper.

(4) In addition, the link between elevation and HDD18, CDD26, and CDD10 was examined using Pearson’s correlation coefficient [45]. The distribution of HDD18, CDD26, and CDD10 in Chongqing was shown using the violin plot [46]. The region represented by each weather station was built using the Thiessen polygon technique, which was developed by Dutch climatologist A.H. Thiessen [47], making sure that each Thiessen polygon only contained one discrete point or weather station.

## 3. Results

### 3.1. Refined Spatial Distribution Characteristics of HDD18, CDD26, and CDD10

**Figure 2:**
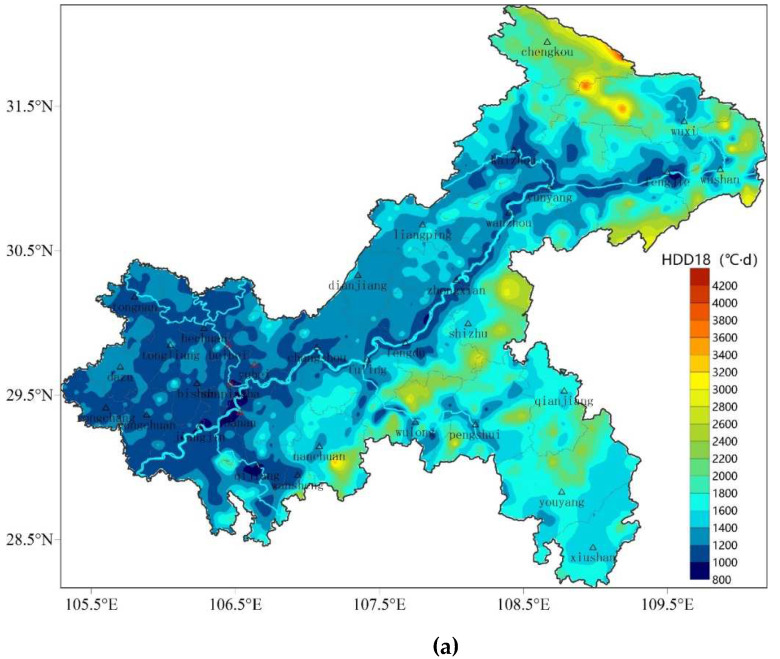

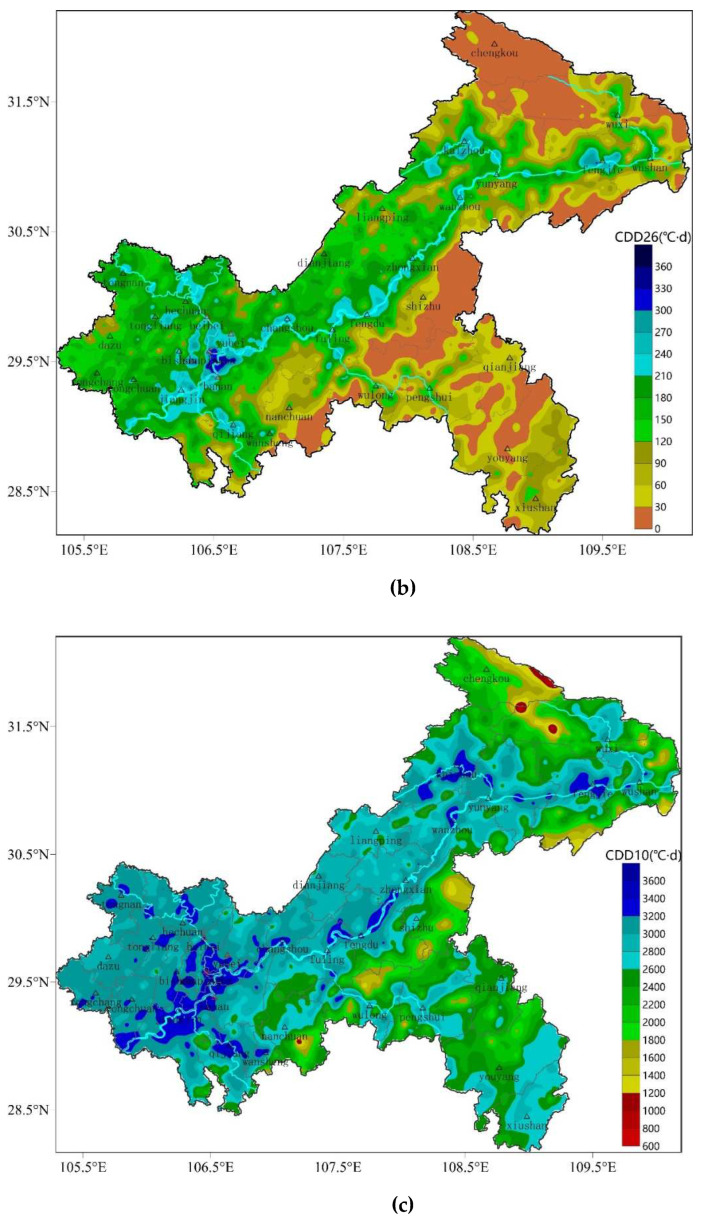
Refined spatial distribution of HDD18 (**a**), CDD26 (**b**), and CDD10 (**c**) in Chongqing (unit: °C·d; the 34 small triangles in the figure indicate the national meteorological stations, among which the 4 small red triangles indicate the national meteorological stations in the main urban area of Chongqing, while the regional meteorological stations are too dense to be marked on the map).

**Figure 3:**
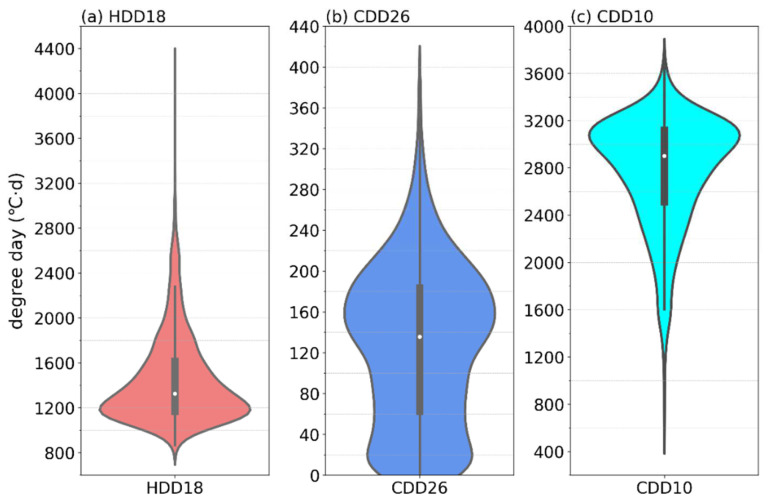
Violin diagram of HDD18 (**a**), CDD26 (**b**), and CDD10 (**c**) in Chongqing (the wider the violin plot, the greater the density; the white dot in the middle of the thick black bar in the middle of the violin plot indicates the median, the top and bottom edges of the thick black bar indicate the upper and lower quartiles, respectively, and the thin black line extending from the thick black bar indicates the 95% confidence interval).

HDD18, which has been widely used in China for building energy efficiency, differs from ASHRAE Standard’s HDD18.3 base temperature by only 0.3 °C. The spatial distribution and magnitude of HDD18 and HDD18.3 are almost identical (see Figure 2a for HDD18 distribution and omitted for HDD18.3 distribution). HDD18 ranges from 871–4224 °C·d, with a high-probability distribution from 1000–1400 °C·d, a median value of 1326 °C·d, a 25% potential value of 1168 °C·d, and a 75% potential value of 1614 °C·d (Figure 3a). HDD18 in Chongqing is in the low to medium value distribution area in China [31,32]. The Yangtze River, Jialing River, and Qijiang River Valley experience the lowest heating degree-days, whereas high-altitude regions in the northeast and southeast experience the most heating degree-days. Other regions experience the second-highest heating degree-days. The spatial distribution characteristics of HDD18 are very similar to those of the topographic distribution in Chongqing (Figure 1). The elevations of the 1908 meteorological stations and HDD18 have Pearson’s correlation values of 0.92, showing a highly substantial positive link.

The indicators CDD26 (in the Chinese standard) and CDD10 (in the ASHRAE Standard) both represent variations in the needs for building cooling. CDD26 ranges from 0 to 387 °C·d in Chongqing, which is a medium- to high-value area in China [31]. The probability distribution of CDD26 ranging from 140 °C·d to 180 °C·d is high, and the probability distribution of CDD26 ranging from 0 °C·d to 20 °C·d is the second highest, with a median value of 136 °C·d, a 25% potential value of 63 °C·d, and a 75% potential value of 183 °C·d (Figure 3b). Since the temperature at some meteorological stations cannot rise above the base temperature of 26 °C, 0 °C·d points appear in CDD26. CDD10 ranges from 590 °C·d to 3684 °C·d in Chongqing, and the probability distribution of CDD10 ranging from 3000 °C·d to 3200 °C·d is high. The median value is 2901 °C·d, the 25% value is 2513 °C·d, and the 75% value is 3118 °C·d (Figure 3c). The regions with the highest CDD values in terms of spatial distribution are those along the Yangtze River, Jialing River, Qijiang River Valley, and Kaizhou. In Chongqing’s main urban region, where there is a very high cooling demand that is partially explained by the urban heat island effect, the deepest blue areas can be seen [48]. While the high-elevation areas in the northeastern and southeastern regions reached the lowest values, the remaining western and central regions had the second-highest values (Figure 2b,c). The Pearson’s correlation coefficients between the heights of the meteorological stations and CDD26 and CDD10 are −0.79 and −0.92, respectively, and the observed inverse correlation is very significant.

### 3.2. Results of Refined Building Climate Zoning in Chongqing Based on the ASHRAE Standard

This section first introduces the regional zoning results based on ASHRAE Standard 169-2013, Standard 169-2020, and Standard 169-2021, respectively, in China. “China climate zones map” can be found in reference [16]. The map is based on 22 years of 1° × 1° average resolution data provided by the NASA Atmospheric Science Data Center from July 1983 to June 2005 (website: http://eosweb.larc.nasa.gov/sse/; accessed on 1 July 2021. some maps show ground station locations superimposed on the climate zone grid). HDD18.3, CDD10, and the monthly mean daily precipitation were calculated and then defined according to ASHRAE climate division schemes (Table 1 and Table 2). The results show that there are two climate zones in the Chongqing area, namely, the 3A (warm-humid) and 4A (mixed-humid) climate zones, of which the area of 4A is larger than 3A. Using the same partitioning method, “China climate zones map” from Standard 169-2020 can be found in reference [17]. The map uses data with a spatial resolution of 0.5° × 0.625° and a temporal resolution of 1 h provided by NASA for 25 years from 1990 to 2014 (website: https://disc.gsfc.nasa.gov/ accessed on 1 July 2021). The findings showed that the Chongqing region has two more climate zones, specifically 3A (warm-humid) and 4A (mixed-humid). The 4A area is primarily found in the southeast and northern northeast of Chongqing, while the 3A area is substantially larger than the 4A area. In reference [18], “China climate zones map” from Standard 169-2021 can be found. The map shows data with a spatial resolution of 0.25° × 0.25° and a temporal resolution of 1 h provided by the European Centre for Medium-Range Weather Forecasts (ECMWF) from 1994 to 2019 (website: https://www.ecmwf.int/en/forecasts/datasets/reanalysis-datasets/era5 accessed on 1 July 2021). The partition results in Chongqing are basically consistent with those depicted in the Standard 169-2020. Via comparison of these three standards, the zoning standards were basically the same. The fineness of the zoning spatial distribution grew from 2013 to 2020 to 2021 as a result of an increase in the spatial resolution of the grid meteorological data employed. Using the cluster analysis method, six national meteorological stations were used in the Chongqing area, and Bai L et al. revised the results of building climate zoning in China based on ASHRAE standard 169-2013 using daily values of meteorological observations from 601 ground-based meteorological stations in China from 1997 to 2013 [22]. “The new building climate zones for China based on the revised definition” can be found in reference [22]. The results showed that the entire area of Chongqing belongs to 3A (Warm-Humid) climate zone. The vast majority of Chongqing is a 3A (Warm-Humid) climate zone type, according to both the Bai et al. [22] study and the ASHRAE’s successive climate zoning standards [16,17,18].

**Figure 4:**
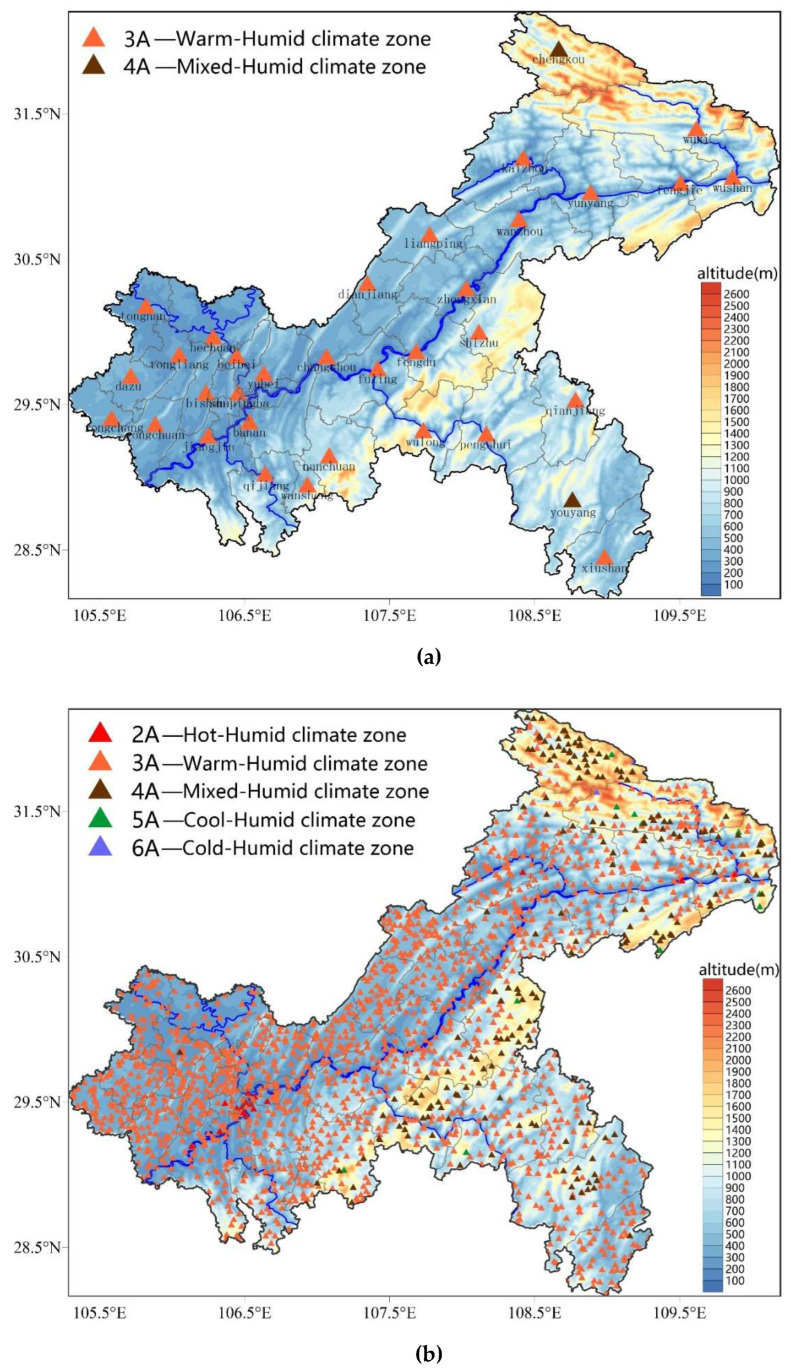
Results of building climate zoning (**a**) and refined climate zoning (**b**) in Chongqing based on the ASHRAE standard.

Based on the ASHRAE standard, the building climate zones in Chongqing were determined using the meteorological stations in the region, and the findings are displayed in Figure 4. First, if only 34 national meteorological stations were used in Chongqing, only two stations, i.e., Chengkou Station in the northeast and Youyang Station in the southeast, belong to the 4A (mixed-humid) type, while the remaining 32 stations belong to the 3A (warm-humid) type (Figure 4a). Then, with the use of high-density observation data from 1908 meteorological stations, the whole region of Chongqing could be divided into five types (Figure 4b): 2A (hot-humid), 3A (warm-humid), 4A (mixed-humid), 5A (cool-humid), and 6A (cold-humid). The 3A type occupied most of Chongqing (1683 meteorological stations, accounting for 88.2% of the total stations), followed by the 4A type (195 meteorological stations, accounting for 10.2% of all stations), which was mainly distributed in the northeastern and southeastern parts of Chongqing, while the 2A type (17 meteorological stations, accounting for 0.9% of the total stations) was largely concentrated in the main urban area of Chongqing, which is closely related to the urban heat island effect [48]. The majority of the type 5A meteorological stations (11 meteorological stations, or 0.6 percent of all stations) were dispersed over an altitude range of 1300–1920 m, with the majority of the stations being in the northeast. The two types of 6A meteorological stations, which make up 0.1% of the total number of stations, are located at site heights of 2047 and 2314 m, which are extremely high and do not matter for Chongqing’s building climate zoning. Building climate zoning is greatly impacted by elevation, as illustrated in Figure 4b. The majority of the type 2A and type 3A meteorological stations were located below 1000 meters above sea level (the number of meteorological stations below 1000 m accounted for 98.8 percent of the total stations). The type 4A, type 5A, and type 6A meteorological stations were mostly located over 1000 m above sea level (69.9% of the stations were above 1000 m), and 25.8% of the type 4A, type 5A, and type 6A stations displayed an altitude between 700 and 1000 m.

In conclusion, the 3A (warm-humid) type filled the majority of the area in the Chongqing building climate zoning results based on the ASHRAE standard, and the corresponding meteorological stations accounted for 88.2 percent of the total stations. A combined 10.9 percent of all meteorological stations were of the 4A (mixed-humid), 5A (cool-humid), and 6A (cold-humid) types. In these regions, there is a need for heating during the winter, and different building design guidelines are applied than in the 2A (hot-humid) and 3A (warm-humid) kinds. The guidance design requirements based on the ASHRAE standard are not discussed here because the existing building climate zoning and architectural design practices in Chongqing rely on Chinese norms.

### 3.3. Refined Climate Zoning Results for Chongqing Building Thermal Design Based on the Chinese National Standard

The Chongqing regional findings from earlier research on building climate zoning in China are reviewed at the outset of this section. Results of the Chinese Building Thermal Design Climate Zones from China’s *Code for thermal design of civil building* (GB50176-2016) can be found in [36]. All of the Chongqing areas were classified as HSCWZ as a consequence of the utilization of four national meteorological stations (Shapingba, Fengjie, Liangping, and Youyang). Based on the results of their utilization of six national meteorological stations in the Chongqing region (Shapingba, Fengjie, Liangping, Youyang, Fuling, and Wanzhou), Bai, Lujian et al. [30,31] also identified the Chongqing region as HSCWZ. “Results of thermal design partitioning of buildings in China” and “New updated thermal climate zones” can be found respectively in reference [30] and [31]. 613 national meteorological stations were used by Liu et al. to study building climate zoning in China between the years 1951 and 1980 and 1981 and 2010; from 1951 to 1980, six national meteorological stations were used in Chongqing (Shapingba, Fengjie, Liangping, Youyang, Fuling, and Wanzhou) [32]. “Climate zoning for buildings in China” can be found in reference [32]. Each of Chongqing’s six stations belonged to the HSCWZ. Five stations in Chongqing belonged to the HSCWZ from 1981 to 2010, and one station (Youyang) belonged to the MZ. In order to analyze building climate zoning across time, Cheng et al. employed 2479 meteorological stations across China between 1961 and 2015, including 34 national meteorological stations in Chongqing [42]. “Spatial distributions of the climate zones for the periods of 1961–1990, 1971–2000, 1981–2010, 1991–2015, respectively” can be found in reference [42]. From 1961 to 1990, 1971 to 2000, 1981 to 2010, and 1991 to 2015, the entire region of Chongqing could be categorized as an HSCWZ. Yang et al. used 701 national meteorological stations in China from 1984 to 2013 to study the application of machine learning based on supervised classification in building climate zoning in China [33]. “Results of building climate zones based on supervised classification method” can be found in reference [33]. The study used 6 national meteorological stations in Chongqing (Shapingba, Fengjie, Liangping, Youyang, Fuling, and Wanzhou), and all 6 Chongqing stations were classified as belonging to zone III, i.e., HSCWZ.

By the end of December 2020, Chongqing held administrative control over 1031 town-level divisions and 38 county-level divisions. However, the national standard of China and earlier research used a very small number of Chongqing meteorological stations, which could have resulted in a low representativeness of the subdivision results. The climatic characteristics of Chongqing’s high-altitude, river valley, and low-altitude central urban districts are very distinct due to the city’s complicated topography. To investigate building thermal design climate zones, it is important to employ more accurate and comprehensive meteorological station data. This strategy could, in large part, address the issue of the underrepresentation of regional climate zones in earlier studies.

**Figure 5:**
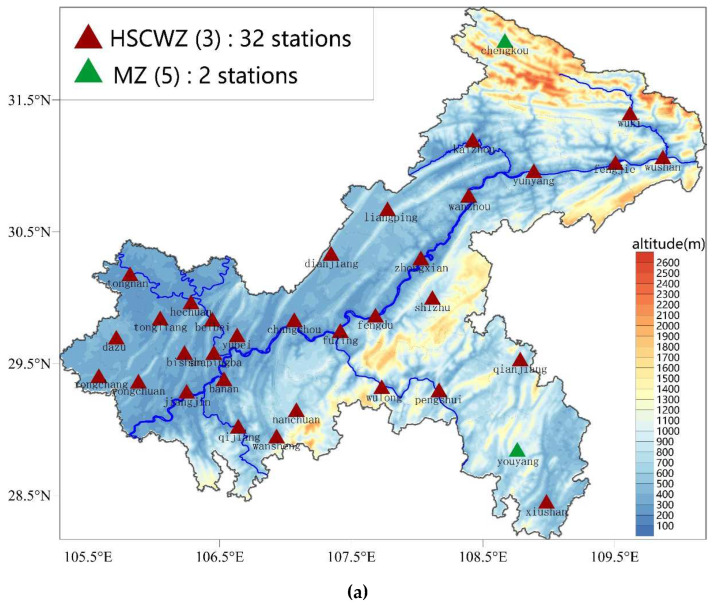

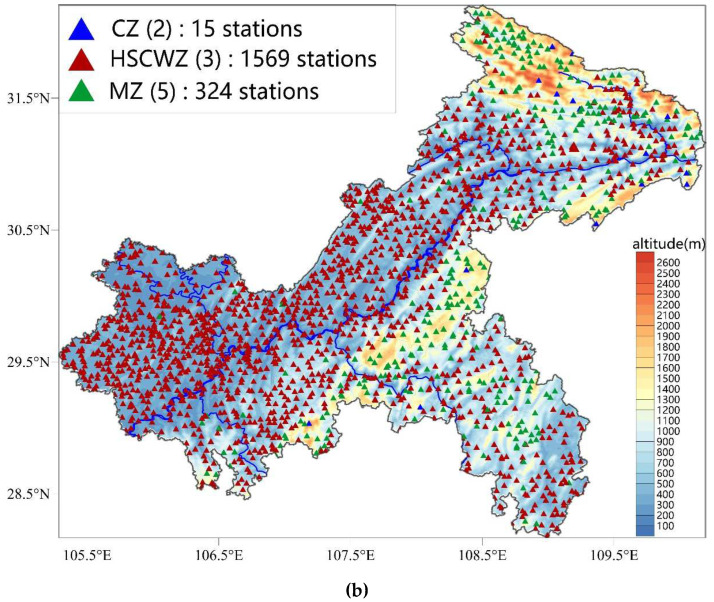
Climate zoning results for building thermal design in Chongqing based on China’s GB50176-2016 ((**a**) Using 34 national meteorological stations, (**b**) Using 1908 meteorological stations).

When only 34 national meteorological stations in Chongqing were used for climate zoning for building thermal engineering design, only two stations, Chengkou in the northeast and Youyang in the southeast, belonged to the mild zone (MZ), and the other 32 stations belonged to the HSCWZ, according to the zoning results based on China GB50176-2016 (Figure 5a); this result is largely consistent with earlier research findings [30,31,32,33,42]. After that, the entire Chongqing area could no longer be categorized as the HSCWZ but could instead be divided into three sorts of areas: CZ, HSCWZ, and MZ based on high-density observation data from 1908 meteorological stations (Figure 5b). CZ areas were mainly distributed in parts of Chengkou, Wuxi, and Wushan in the northeast; HSCWZ areas largely occurred in the main urban area, most of the western, southwestern, and central parts, and parts of the northeast and southeast, and MZ areas were mostly found in parts of the northeast and southeast. Figure 5b demonstrates that altitude has a major impact on climatic zoning for building thermal design, similar to Figure 4b. The majority of HSCWZ regions were found below 1000 m in altitude, MZ regions were found between 1000 and 1600 m, and CZ regions were found above 1600 m. As altitude rises, the temperature progressively drops, the need for cooling energy declines, and the need for heating energy rises. As a result, the climate design of buildings gradually switches from cooling to heating. Regarding the distribution of meteorological stations, there were 15 stations in the CZ, making up 0.8% of all stations, 1569 stations in the HSCWZ, making up 82.28% of all stations, and 324 stations in the MZ, making up 17.0% of all stations. The 1908 meteorological stations are discrete points. Then, using the Thiessen polygon algorithm, the area and percentage of these three different sorts of regions were calculated. In comparison, the MZ area made up 22.7 percent of the whole area, the CZ area included 1.5 percent of the total area, and the HSCWZ area made up 75.8 percent of the total area. The percentage of meteorological stations and locations in the specialized climate zones for the Chongqing building thermal engineering design are shown in Table 3.

To ensure adaptation to local conditions, thermal design climate zones were created. Different building climate zones have different architectural design requirements, which means that buildings must be able to fully utilize and be adaptable to different climate conditions. The building thermal design climate of Chongqing can be categorized into three types in this section: CZ, HSCWZ, and MZ, as per the primary zoning indicators of the national standard of China. Each type exhibits corresponding design requirements such as summer cooling and winter heating [36]. Therefore, selecting the proper model requires taking into account the variety of climate conditions across various subdivisions.

Shi Q et al. showed that scientific and reasonable building climate zoning will have a positive effect on energy saving and emission reduction in the building sector [49]. China’s national standard “*General code for energy efficiency and renewable energy application in buildings* (GB 55015-2021)”, which was implemented from 1 April 2022, specifies in detail the average energy consumption indexes of buildings in different building climate zones, and here we only take the average energy consumption indexes of heating, cooling, and lighting in new public buildings in this standard as an example [44]. “Table of Average energy consumption index for heating, cooling, and lighting of all kinds of new public buildings” can be found in reference [44]. We initially estimated the energy saving percentage of building heating, cooling, and lighting energy consumption in new public buildings after adopting the refined building climate zoning of this study in Chongqing. Estimation method: energy saving percentage = [Building energy consumption based on the *Code for thermal design of civil buildings* (GB 50176-2016)—Building energy consumption based on refined climate zoning design]/Building energy consumption based on the *Code for thermal design of civil buildings* (GB 50176-2016) × 100%. The preliminary estimation results show that for new public buildings, according to different building categories, office buildings with floor area < 20,000 m^2^ will save 6.8%, office buildings with floor area ≥ 20,000 m^2^ will save 5.7%, hotel buildings with floor area < 20,000 m^2^ will save 6.8%, hotel buildings with floor space greater than or equal to 20,000 m^2^ will save 3.3%, commercial buildings will save 7.9%, hospital buildings will save 8.1%, and school buildings will save 2.4%. This example shows that refined building climate zoning will make a positive contribution to building energy efficiency and emission reduction in Chongqing to a certain extent. However, the research focus of this paper is refined zoning of building climate. The possible influence of refined zoning of building climate on building emission reduction is only briefly analyzed here, and detailed analysis will be conducted in another paper.

## 4. Conclusions

This article examined climate zoning for building thermal design in the Chongqing region of China based on ASHRAE standards and China’s GB50176-2016 using daily data from 1908 intense surface meteorological stations from 2011 to 2020. The findings include the following:

(1) Chongqing’s entire territory is classified into five different types of climate zones, according to the findings of revised building climate zoning conducted there using ASHRAE standards. Type 3A (warm-humid) meteorological stations occupied the majority of the area, accounting for 88.2% of them. Type 4A (mixed-humid), Type 5A (cool-humid), and Type 6A (cold-humid) meteorological stations jointly accounted for 10.9% of all stations. In comparison to other areas, the areas of 4A, 5A, and 6A have more heating degree days than cooling degree days, indicating that these areas have lower summer cooling demand and some winter heating needs. Building design guidelines for 4A, 5A, and 6A zones differ from those for 2A (Hot-Humid) and 3A types.

(2) The entire region of Chongqing is split into three types of areas: CZ, HSCWZ, and MZ, according to the refined climate zoning results of the building thermal design utilizing China’s GB50176-2016. In CZ, the meteorological stations occupy 0.8% and 1.5% of the total area. In HSCWZ, the meteorological stations occupy 75.8% and 82.2% of the total area. The meteorological stations in MZ account for 17.0% and 22.7% of the area. MZ are primarily situated between 1000 and 1600 meters above sea level in Chongqing’s northeastern and southeasterly regions. Preliminary projections indicate that refined building climate zoning will contribute positively to building energy efficiency and emission reduction in Chongqing, taking the building heating, cooling, and lighting energy consumption of new public buildings as an example.

## 5. Discussion

The requirements for building climate design vary for each climate zone under the pertinent building energy efficiency standards. The results of this study for the building climate zoning of Chongqing, China, using ASHREA Standard are more precise than the results for the Chongqing region utilizing ASHREA Standard 169-2013 [16], 169-2020 [17], and 169-2021 [18]. In particular, Type 4A (Mixed-Humid), Type 5A (Cool-Humid), and Type 6A (Cold-Humid) locations in this study are differentiated from Type 2A (Hot-Humid) and Type 3A (Cool-Humid) by the pertinent criteria used to guide building design.

According to the results based on China’s GB50176-2016 and previous studies [30,32,33,42], the whole Chongqing area belonged to the HSCWZ. However, the terrain in the Chongqing area is complicated, and the three-dimensional climate characteristics are significant. According to China’s GB50176-2016 building thermal design climatic refinement zoning, the entire territory of Chongqing was separated into CZ, HSCWZ, and MZ for this study. Not to be overlooked in particular is the MZ, which is primarily found at higher elevations in the southeast and northeastern regions of Chongqing. The building energy efficiency guidelines and tactics used in the MZ are significantly different from those used in the HSCWZ [44,50,51].

Last but not least, this research case supports regional climate adaption, energy conservation, and emission reduction in green buildings by helping to develop and revise regional building energy efficiency standards. This research example is appropriate for dissemination and application in other areas with challenging topography. However, the next study will take into account administrative divisions at the township level, in addition to meteorological conditions when improving and enforcing building energy efficiency regulations.

## Figures and Tables

**Table 1:** Definitions of climate types in the ASHRAE standards [16,17,18].

Climate Zone Definition	Definition
Humid zone (A)	Locations that are not marine (C) and not dry (B).
Dry zone (B)	(1) Not marine; (2) If 70% or more of precipitation *p* occurs during the high-sun period, the dry/humid threshold is: *p* < 20 × (T + 14); (3) If between 30% and 70% of precipitation *p* occurs during the high-sun period, then the dry/humid threshold is: *p* < 20 × (T + 7); (4) If 30% or less of precipitation *p* occurs during the high-sun period, the dry/humid threshold is: *p* < 20 × T.
Marine zone (C)	(1) Mean temperature in the coldest month varies between −3 °C and 18.3 °C; (2) Warmest monthly mean < 22 °C; (3) At least four months with a mean temperature over 10 °C; (4) Dry season in summer. The month with the highest precipitation in the cold season has at least three times as much precipitation as the month with the least precipitation in the rest of the year.
Remarks: (1) *p* = annual precipitation (mm); (2) *T* = annual mean temperature (°C); (3) Summer or high-sun period ranges from April through September in the Northern Hemisphere and from October through March in the Southern Hemisphere; (4) Winter or cold season ranges from October through March in the Northern Hemisphere and from April through September in the Southern Hemisphere.	

**Table 2:** Thermal climate zone definitions of the ASHRAE [16,17,18].

Thermal Zone	Zone Name	Zoning Criterion
0	Extremely hot	6000 < CDD10
1	Very hot	5000 < CDD10 ≤ 6000
2	Hot	3500 < CDD10 ≤ 5000
3	Warm	CDD10 ≤ 3500 and HDD18.3 ≤ 2000
4	Mixed	CDD10 ≤ 3500 and 2000 < HDD18.3 ≤ 3000
5	Cool	CDD10 ≤ 3500 and 3000 < HDD18.3 ≤ 4000
6	Cold	4000 < HDD18.3 ≤ 5000
7	Very cold	5000 < HDD18.3 ≤ 7000
8	Subarctic/arctic	7000 < HDD18.3

**Table 3:** Proportions of the meteorological stations and area in climate zoning for refined building thermal design climate zoning in Chongqing based on China’s GB50176-2016.

Climate Zones	Number of Meteorological Stations	Proportion of Meteorological Stations	Proportion of the Area
CZ	15	0.8%	1.5%
HSCWZ	1569	82.2%	75.8%
MZ	324	17.0%	22.7%

## Data Availability

Not applicable.

## Nomenclature

ASHRAE	American Society of Heating, Refrigerating and Air Conditioning Engineers
CDD26	Cooling degree-days based on 26 °C
CDD10	Cooling degree-days based on 10 °C
CZ	Cold zone
ECMWF	European Centre for Medium-Range Weather Forecasts
HDD18	Heating degree-days based on 18 °C
HDD18.3	Heating degree-days based on 18.3 °C
HSCWZ	Hot summer and cold winter zone
HSWWZ	Hot summer and warm winter zone
MZ	Mild zone
NASA	National Aeronautics and Space Administration
SCZ	Severe cold zone
SCS	Summer climatic severity
T_1_	Average air temperature in January (°C)
T_7_	Average air temperature in July (°C)
WCS	Winter climatic severity
